# New Year’s Greeting

**Published:** 1859-01

**Authors:** 


### HALL'S JOURNAL OF HEALTH.

OUR LEGITIMATE SCOPE IS ALMOST BOUNDLESS: FOR WHATEVER BEGETS PLEASURABLE AND HARMLESS FEELINGS, PROMOTES HEALTH; AND WHATEVER INDUCES DISAGREEABLE SENSATIONS, ENGENDERS DISEASE.

*We aim to show how Disease may be avoided, and that it is best, when sickness comes, to take no Medicine without consulting an educated Physician.*

VOL. VI.] JANUARY, 1859. [No. 1.

### NEW YEAR'S GREETING.

The commencement of another year and a new volume, offers a fit occasion for the expression of our thanks for the kindly partiality with which our journal has been received by an indulgent press and public, in that our enterprise has been a success from the beginning, having been encouragingly profitable directly and indirectly, still more highly so; hence we feel ourself under obligation to endeavor to make it more worthy of the universal appreciation in which it has been held. To this end, we have arranged to turn over everything connected with the publishing department to H. B. Price, a native of our city, known to every publishing house of any age in New York, as a gentleman of enterprise, energy and business promptitude, with an integrity which must command respect and ensure success. All letters therefore which pertain to dollars and cents, yearly subscriptions, or separate numbers, should be addressed to "Hall's Journal of Health, New York." All letters pertaining to the editorial department, or for professional advice, should be directed to Dr. W. W. Hall, New York. And while our indefatigable publisher gathers himself up to the work of doubling our circulation for eighteen hundred and fifty-nine, as good old fifty-eight did on fifty-seven, the editor, with a right good will and a merry heart, lays off his coat and takes up his pen for the labors of another year, trusting that as to his patrons, it will add no gray hair, deepen no furrow, and bring no tear, unless they be such as will in their influences on the character and heart, the better prepare them for that more blessed world, where tears fall not and sickness never comes; where we shall be always well, always young, always good.

